# Systematic review of therapy used in relapsed or refractory diffuse large B-cell lymphoma and follicular lymphoma

**DOI:** 10.4155/fsoa-2018-0049

**Published:** 2018-07-19

**Authors:** Aaron Galaznik, Rachel Huelin, Michael Stokes, Yelan Guo, Meredith Hoog, Tarun Bhagnani, Jill Bell, Yaping Shou

**Affiliations:** 1 Millennium Pharmaceuticals, Inc., a wholly owned subsidiary of Takeda Pharmaceutical Company Limited, 40 Landsdowne Street, Cambridge, MA 02139, USA; 2 Meta Research, Evidera, 500 Totten Pond Road, Fifth Floor, Waltham, MA 02451, USA; 3 Data Analytics, Evidera, 7575 Trans-Canada Highway, Suite 404, St-Laurent, Quebec H4T 1V6, Canada; 4 Meta Research, Evidera, Metro Building, 6^th^ Floor, 1 Butterwick, London W6 8DL, United Kingdom; 5 Modelling and Simulation, Evidera, 7101 Wisconsin Avenue, Suite 1400, Bethesda, MD 20814, USA; 6 Data Analytics, Evidera, 500 Totten Pond Road, Fifth Floor, Waltham, MA 02451, USA

**Keywords:** chemotherapy, DLBCL, FL, overall survival, refractory, relapse, response, rituximab, stem cell therapy, systematic literature review

## Abstract

To identify real-world evidence on outcomes from therapies for relapsed/refractory diffuse large B-cell lymphoma (DLBCL) and follicular lymphoma (FL), we systematically reviewed literature in Medline/Embase for DLBCL/FL-related articles on real-world results published during January 2012–May 2016. Among 33 included articles, therapies included stem cell transplant (SCT) and chemotherapy, including experimental regimens. The highest overall survival rates were observed for SCT, long considered an optimal strategy following initial relapse. Prognoses were inferior among DLBCL patients receiving rituximab-based regimens rather than SCT, particularly among studies that exclusively focused on those ineligible for SCT due to age or co-morbidity. A lack of viable treatment options for DLBCL/FL patients ineligible for SCT after relapse remains a significant gap in care.

## Lay abstract

Non-Hodgkin lymphoma is the most prevalent blood cancer. Diffuse large B-cell lymphoma and follicular lymphoma account for nearly two-thirds of all non-Hodgkin lymphomas. One-third of patients with diffuse large B-cell lymphoma continue on to relapsed or refractory disease. While follicular lymphoma tends to be less aggressive, relapses do occur. Stem cell transplant and chemotherapy/immunotherapy are the current treatment options for relapsed or refractory patients. However, many patients are ineligible for stem cell transplant, due to age or preexisting medical conditions, so safe and effective treatment choices for these patients are a must. The lack of viable treatment options highlights this unmet need.

Non-Hodgkin lymphoma (NHL) is the most prevalent hematological malignancy and consists of a heterogeneous group of lymphoproliferative malignant diseases, characterized by a high degree of variety in pathology and clinical features. It is estimated that 85–90% of NHLs originate from B cells, and the remaining NHLs have a T-cell and natural killer cell lineage [1]. Diffuse large B-cell lymphoma (DLBCL) and follicular lymphoma (FL), both derived from B cells, are the two most common types of NHL, accounting for approximately 65% of all NHLs [1].

DLBCL is the most common of the NHLs and it is estimated to account for up to 40% of all NHLs [2]. Incidence of DLBCL increases with age and outcomes are variable. The National Comprehensive Cancer Network (NCCN) International Prognostic Index (IPI) is a scoring system that is used to assign patients to one of four different risk groups at the time of diagnosis – low, low-intermediate, high-intermediate and high [3,4]. This scoring system includes five predictors: age, lactate dehydrogenase levels, sites of involvement, Ann Arbor stage and Eastern Cooperative Oncology Group Performance Status. Based on the NCCN-IPI, 5-year overall survival (OS) rates range from 96% in low-risk patients to 33% in high-risk patients [3].

DLBCL exhibits a high degree of genetic, clinical and histologic variability; however, this observation has not been translated into current treatment algorithms [2,3]. The current treatment for DLBCL is R-CHOP – rituximab (R), a CD-20-directed monoclonal antibody, given in combination with CHOP, the standard chemotherapeutic regimen of cyclophosphamide, doxorubicin, vincristine and prednisone [3]. The number and timing of R-CHOP cycles may differ depending on whether the disease is localized or advanced. The addition of rituximab to the standard CHOP treatment regimen has had a significant effect on survival outcomes, improving them by approximately 15% [2]. Despite an overall good prognosis for patients diagnosed with DLBCL, approximately a third of patients will develop relapsed or refractory (R/R) disease. Treatments for R/R DLBCL patients include autologous stem cell transplant (auto-SCT) and chemotherapy/immunotherapy; however, there is limited clinical evidence on the real-world outcomes of these treatments. For patients who are candidates for auto-SCT and have chemosensitive disease, salvage chemotherapy followed by high-dose chemotherapy with auto-SCT is recommended. This has been shown to be effective in the long-term control of the disease in approximately half of these patients [2]. For patients who are not auto-SCT candidates due to age or co-morbidities, treatment with other chemotherapeutic agents with or without rituximab is recommended; however, prognosis for these patients is poor. Patients with progressive disease after three successive treatment regimens also have a poor prognosis, and are unlikely to derive any further benefit from currently available therapies [2,3].

FL is the most common of the indolent NHLs and accounts for approximately 22% of all NHLs [3]. Most patients are approximately 50 years of age at diagnosis, at which point the disease is often already widespread [5]. Median survival ranges from 8 to 15 years with 5-year survival outcomes ranging from 52.5 to 91%. Three prognostic groups have been defined by the Follicular Lymphoma International Prognostic Index, a prognostic scoring system that is based on age, Ann Arbor stage, number of nodal sites involved, hemoglobin levels and serum lactate dehydrogenase levels [2]. Guidelines specifically for treating FL are based on Grade, and mainly apply to patients with Grade 1 or Grade 2 disease. Currently, the distinction between Grades 3A and 3B has not been shown to be clinically significant; FL Grade 3A may be treated as FL by some clinicians and as DLBCL by others [3]. However, FL Grade 3B is commonly treated as DLBCL. Initial treatment for FL includes involved-site radiotherapy. An alternative to involved-site radiotherapy is immunotherapy, with or without chemotherapy, with or without radiotherapy [3]. For patients who relapse or who have progressive disease, hematopoietic SCT (autologous and allogeneic) can lead to an increase in progression-free survival (PFS) and OS [1,3]. Histological transformation of FL to DLBCL is generally associated with a poor clinical outcome, with a median OS of less than 2 years [2].

Since the addition of rituximab to the CHOP regimen approximately 15 years ago, there have been no additional approvals for any novel front-line therapies for either DLBCL or FL, nor any meaningful improvements to the R-CHOP survival rates [6]. With approximately a third of DLBCL patients experiencing R/R disease, many of whom are ineligible for SCT, there exists an unmet need, and the development of more effective therapies for these patients remains an important objective. Overall, while much has been published about NHL in general, there is a lack of clinical evidence specific to patients with R/R DLBCL or R/R FL. Furthermore, there is limited knowledge regarding healthcare utilization and the economic burden of these two diseases. Therefore, this review was designed to systematically collect and review information to evaluate the real-world evidentiary base for outcomes associated with therapies used to treat R/R DLBCL or R/R FL.

## Methods

This systematic literature review was conducted according to the Preferred Reporting Items for Systematic Reviews and Meta-Analyses (PRISMA) guidelines. Using a prospectively established protocol, a systematic search was conducted in MEDLINE (via PubMed) and Embase to identify studies that report real-world treatment outcomes in patients with DLBCL and FL published from 1 January 2012 to 11 May 2016. We also reviewed conference abstracts for the two most recent meetings (as of June 2016) of the following groups: American Society of Clinical Oncology, European Society for Medical Oncology, American Society of Hematology, European Hematology Association and International Society for Pharmacoeconomics and Outcomes Research.

In the first round of screening, all titles and abstracts were screened by a single investigator against the inclusion and exclusion criteria, using the PICOS-T elements (participants, interventions, comparisons, outcomes, study design and time period; Table 1). Although the focus of this manuscript is R/R DLBCL/FL, the patient population queried at this stage was adult patients with DLBCL or FL. A senior investigator validated 10% of the rejected abstracts to confirm accuracy. No study was excluded at the abstract level based solely on insufficient information. Full-text articles for the accepted abstracts were retrieved for in-depth review in the second round of screening, conducted by a single investigator using the same inclusion and exclusion criteria applied at the abstract level. Only studies enrolling ≥50 patients were included, and a geographic emphasis was placed on studies conducted primarily in France, Germany, Italy, Spain, the United Kingdom, the USA and Japan. A second investigator confirmed all excluded studies; any discrepancies were resolved by a third investigator. Throughout the process, discrepancies were addressed by consensus and with input from a third investigator if necessary.

**Table 1.** **Study selection criteria (participants, interventions, comparisons, outcomes, study design and time period).**

**Criteria**	**Inclusion criteria**
Population(s)	Adult patients (≥ 18 years) with diffuse large B-cell lymphoma or follicular lymphoma

Interventions	Chemotherapeutic/immunotherapy agents licensed and under investigation for these conditions, where applicable

Comparisons	Not applicable

Outcomes	• Effectiveness: treatment response rates (cytogenetic and hematologic responses), duration of response, progression-free survival, overall survival • Tolerability/safety: overall/severe AEs, withdrawals, Grade 3 to 4 AEs • Economic: ICERs, QALYs, life-years gained, costs and healthcare resource use • HRQoL/patient-reported outcomes/utilities: patient-reported outcomes, HRQoL outcomes (measured via generic [e.g., EQ-5D] or disease-specific instruments [e.g., EORTC QLQ-C30]) • Treatment patterns and guidelines

Time	Indexed database: 1 January 2012 to 10 May 2016 Gray literature: Two most recent meetings

Study design	• Clinical effectiveness and safety, treatment patterns: observational studies • Economic evaluations: economic analyses (e.g., CEMs, CBAs, CUAs, CMAs) • HRQoL/patient-reported outcomes/utilities: all study designs • Costs and resource use: all study designs • Clinical guidelines

Other	English language only; Geographic emphasis on the USA, France, Germany, Italy, Spain, United Kingdom and Japan

AE: Adverse event; CBA: Cost–benefit analysis; CEM: Cost–effectiveness model; CMA: Comparative market analysis; CUA: Cost–utility analysis; EORTC QLQ-C30: European Organisation for Research and Treatment of Cancer Quality of Life Questionnaire-C30; EQ-5D: EuroQol five dimensions questionnaire; HRQoL: Health-related quality of life; ICER: Incremental cost–effectiveness ratio; QALY: Quality-adjusted life-year.

The literature search included publications (English only) on randomized controlled trials (RCTs), observational studies and economic evaluations that reported clinical effectiveness, tolerability/safety, economic or health-related quality of life/patient reported outcomes in adult patients with DLBCL or FL.

Full data extraction was performed on all studies included following the second round of screening. Extracted data included study descriptors; patient characteristics; treatment-level information; outcomes (efficacy, safety/tolerability, economic, health-related quality of life and patient reported outcomes); treatment patterns and clinical guidelines.

## Results

**Figure 1.**
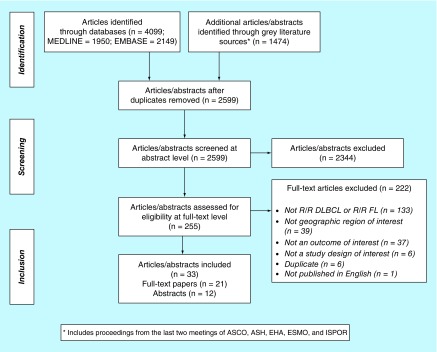
**The Preferred Reporting Items for Systematic Reviews and Meta-Analyses study attrition diagram.** ASCO: American Society of Clinical Oncology; ASH: American Society of Hematology; DLBCL: Diffuse large B cell lymphoma; EHA: European Hematology Association; ESMO: European Society for Medical Oncology; FL: Follicular lymphoma; ISPOR: International Society for Pharmacoeconomics and Outcomes Research.

At the abstract level, a total of 2599 unique citations were identified. After these abstracts were screened, 255 had a sample size of ≥50 patients and were selected for full-text review; 2344 were excluded. The main reasons for exclusion during the full-text review were the patient population not meeting selection criteria (e.g., no R/R DLBCL or R/R FL patients included or mixed populations), the article not reporting data on a treatment-related effectiveness outcome (n = 37) or the study enrolling patients outside of the review's geographical scope (n = 39). After applying all criteria, 33 articles [7–39] examining either efficacy or effectiveness of therapies administered to patients with R/R DLBCL or R/R FL were identified. The PRISMA study attrition diagram is shown in Figure 1.

Of the 33 included studies, 21 were full-text papers and 12 were scientific conference abstracts. There were 18 articles reporting on patients with R/R DLBCL and 15 reporting on R/R FL. The majority of publications had observational study designs (n = 21).

### Summary of studies by treatment & patient characteristics

Of the 18 studies evaluating patients with R/R DLBCL, SCT was the most common treatment, examined in seven articles. Six studies looked at rituximab-based chemo, three at experimental chemo and two discussed carmustine (BCNU), etoposide, Ara-C and melphalan (BEAM) conditioning. Experimental chemotherapy was defined as any drug regimen that did not include rituximab, or that was investigated as part of a clinical trial. Most publications reported data on disease stage, and enrolled patients across all stages. Proportions of patients with Stage 3 or 4 DLBCL ranged from 31 to 76%. In studies of R/R FL, the majority of articles examined SCT (n = 9), followed by experimental chemotherapy (n = 3), Yttrium-90 Ibritumomab Tiuxetan (90Y-IT; n = 1), watchful waiting (n = 1) and salvage treatment (n = 1).

### Clinical outcomes of treatment for R/R DLBCL

#### Observational study designs

Twelve of the studies examining R/R DLBCL were of an observational study design (Table 2). Of these, the majority (7/12) investigated the effectiveness of either autologous (auto-) SCT or allogeneic (allo-) SCT, and reported on OS; OS ranged from 18% at 3 years for the subset of patients with non-germinal center B-cell-like DLBCL, to 64% at 4 years in an overall population of R/R DLBCL [8,11,14,15,20,21,26]. One study [21] directly compared auto-SCT with allo-SCT and found that myeloablative conditioning used prior to allo-SCT was significantly inferior to auto-SCT for both OS (hazard ratio [HR]: 1.9; 95% CI: 1.5–2.4) and PFS (HR: 1.4; 95% CI: 1.1–1.7) outcomes, after adjusting for baseline and demographic characteristics. However, for reduced-intensity conditioning (RIC) allo-SCT, the OS disadvantage (HR: 1.3; 95% CI: 1.0–1.8) was of only borderline significance, and the disadvantage for PFS was not significant (HR: 1.2; 95% CI: 0.9–1.6). Another study, using data from the European Group for Blood Marrow Transplantation database, compared unrelated donor allo-SCT with allografts from sibling hematopoietic SCT [8]. At 3 years, neither OS (42 vs 37% for sibling and unrelated donors, respectively) nor PFS (35 vs 35% for sibling and unrelated donors, respectively) was associated with donor type. This was confirmed in multivariate analyses. Among all of the studies investigating SCT, the highest OS rate of 71% was reported in a study examining auto-SCT at a median follow-up of 39 months [9]. This study also found that patients who relapsed as a result of chemosensitivity had higher 3-year event-free survival (EFS; 66%) compared with refractory patients (35%, p = 0.001).

**Table 2.** **Summary of observational studies examining clinical outcomes of treatment for relapsed or refractory diffuse large B-cell lymphoma.**

**Country or region**	**Design**	**Treatment setting**	**Sample size**	**Follow-up (months)**	**Survival/progression**	**Response**	**Study (year), Ref.**	**Source**
France, Germany, Italy, Spain, UK	Retrospective cohort	Auto-SCT vs Allo-SCT	6717	Median 18 mos (among survivors)	OS at 4 yrs: Auto-SCT: 54% MAC-allo-SCT: 30% RIC-allo-SCT: 38% PFS at 4 yrs: Auto-SCT: 43% MAC-allo-SCT: 28% RIC-allo-SCT: 28%	NR	Robinson *et al*. (2016), [21]	Full text

France, Germany, Italy, Spain, UK	Retrospective cohort	Allo-SCT: unrelated donor vs sib-SCT	473	Median 45 mos (among survivors)	OS at 3 yrs: Sibling: 42% Unrelated: 37% PFS at 3 yrs: Sibling: 35% Unrelated: 35%	NR	Avivi *et al*. (2014), [8]	Full text

France, Germany, Italy, Spain, UK	Retrospective cohort	Auto-SCT	86	Median 39 mos	OS: 71% at median follow-up of 39 months EFS at 3 yrs: Chemosensitive: 66% Refractory: 35% Transplant in complete or partial response: 100%	NR	Avivi *et al*. (2014), [9]	Abstract

USA	Observational	Allo-SCT after prior auto-SCT	503	Median 55 mos (among survivors)	OS at 3 yrs: Overall: 37% Low risk: 43% Intermediate risk: 25% High risk: 14% PFS at 3 yrs: Overall: 31%; Low risk: 38% Intermediate risk: 19% High risk: 10%	NR	Fenske *et al*. (2015), [11]	Full text

Italy	Retrospective cohort	Allo-SCT after prior auto-SCT	165	Median 39 mos (among survivors)	OS 1 yr: 55% 3 yrs: 42% 5 yrs: 39% PFS 1 yr: 48% 3 yrs: 34% 5 yrs: 31%	ORR: 49% CR: 65% (among evaluable patients)	Rigacci *et al*. (2012), [20]	Full text

USA	Retrospective cohort	Allo-SCT in patients with relapse/progression after prior chemo	Overall: 101 PMBL: 17 GBC: 62 Non-GBC: 22	Median mos PMBL: 52 GBC: 63 Non-GBC: 29	OS at 3 yrs: PMBL: 46% GBC: 52% Non-GBC: 18% PFS at 3 yrs: PMBL: 41% GBC: 39% Non-GBC: 12%	NR	Khouri *et al*. (2014), [26]	Abstract

USA	Retrospective cohort	Allo-SCT in patients with relapse/progression after prior chemo	53	Median 56 mos	OS at 4 yrs: Overall: 64% CTD positive: 38% CTD negative: 67% PFS at 4 yrs: Overall: 46% CTD positive: 13% CTD negative: 48%	NR	Herrera *et al*. (2015), [14]	Abstract

Italy	Retrospective cohort	Rituximab-based regimens: rituximab + bendamustine	55	Median 10.6 mos	Median OS: 10.8 mos Median PFS: 8.8 mos	ORR: 50% CR: 28% DOR: 20.3 mos (among patients with PR or CR)	Arcari *et al*. (2015), [7]	Full text

USA	Prospective study	Rituximab-based regimens: rituximab + CHOP	258	Median 59 yrs	Median OS Responsive disease (CR or PR): 21.0 mos Stable or progressive disease: 6.8 mos PFS at 2 yrs: 45% (post-transplant)	NR	Farooq *et al*. (2015), [10]	Abstract

France, Germany, Italy, Spain, UK	Retrospective cohort	Rituximab-based regimens: Rituximab added as salvage therapy	144	Median 6.5 yrs (among survivors)	OS, median from relapse was 1.12 yrs for R/R CR patients 5-yr survival from progression: Primary chemorefractory (1991–2001): 6% Primary chemorefractory (2002–2012): 15%; Relapsed (1991–2001): 33% Relapsed (2002–2012): 42%	CR: Primary chemorefractory (1991–2001): 3% Primary chemorefractory (2002–2012): 17% Relapsed (1991–2001): 51% Relapsed (2002–2012): 59% PR: Primary chemorefractory (1991–2001): 2% Primary chemorefractory (2002–2012): 26% Relapsed (1991–2001): 39% Relapsed (2002–2012): 26%	Rovira *et al*. (2015), [24]	Full text

USA	Retrospective cohort	Beam conditioning: BEAM/R vs Z-BEAM	Overall: 113 Group A: 57 Group B: 26 Group C: 16 Group D: 14	Median yrs Group A: 11.8 Group B: 8.1 Group C: 4.2 Group D: 4.9	OS at 5 yrs Group A: 74% Group B: 73% Group C: 69% Group D: 86% DFS at 5 yrs Group A: 62% Group B: 65% Group C: 63% Group D: 63%	NR	Khouri *et al*. (2015), [15]	Abstract

Germany	Retrospective cohort	Modified R-DHAP regimen	122	NR	NR	ORR: 74%, CR: 17%, PR: 32%	Lisenko *et al*. (2015), [25]	Abstract

Auto/allo-SCT: Autologous/allogeneic stem cell transplantation; BEAM: Etoposide, Ara-C and melphalan; CHOP: Cyclophosphamide, doxorubicin, vincristine and prednisone; CR: Complete response; CTD: Circulating tumor DNA; DFS: Disease-free survival; DOR: Duration of response; EFS: Event-free survival; GBC: Germinal center B-cell-like; MAC: Myeloablative conditioning; mos: Months; NR: Not reported; ORR: Overall response rate; OS: Overall survival; PFS: Progression-free survival; PMBL: Primary mediastinal B-cell lymphoma; PR: Partial response; R-DHAP: Rituximab dexamethasone, cytarabine and cisplatin; RIC: Reduced-intensity conditioning; R/R: Relapsed/refractory; SCT: Stem cell transplant; sib-SCT: Sibling stem cell transplantation; yrs: Years.

The remainder of the observational studies reported on outcomes in R/R DLBCL patients treated with either rituximab-based regimens (n = 3) or BEAM conditioning (n = 1). The studies investigating rituximab-based regimens reported median OS ranging from 10.8 to 21.0 months [7,10]. Farooq *et al*. showed that patients in complete response at the end of first-line therapy have a significant survival advantage relative to those with stable disease or partial response (median OS: 21 vs 6.8 months, respectively) [10]. A study examining longer-term survival of 5 years from progression found that primary chemorefractory patients had lower OS (15%) compared with relapsed patients (42%) [24]. In the one observational study evaluating the effectiveness of BEAM conditioning regimens [15], 5-year OS rates showed a slight advantage for Z-BEAM over BEAM/R (86 vs 69%) during the 2007–2010 period. However, no difference in the rate of 5-year disease-free survival (DFS) was observed.

There were limited data on response outcomes. Response rates were reported in only three of the observational studies identified. Overall response rates (ORRs) of 49% were reported in a study examining allo-SCT after prior auto-SCT; this was the only SCT study reporting data on response outcomes [20]. In another study, an ORR of 50% and complete response of 28% were reported for R/R DLBCL patients treated with rituximab + bendamustine [7]. The article by Rovira *et al*. [24] also compared complete response outcomes in primary chemorefractory and relapsed patients. As with OS, the authors demonstrated a significant advantage with respect to complete response for relapsed patients (59 vs 17% for relapse vs chemorefractory during the 2002–2012 period).

#### Clinical trials

Six publications discuss trials for R/R DLBCL treatments (Table 3). Three clinical trials examined the efficacy of experimental chemotherapy regimens. Czuczman *et al*. compared lenalidomide with investigator's choice regimen (gemcitabine, rituximab, etoposide or oxaliplatin) and found that median OS (31 vs 24.6 weeks), median EFS (13.6 vs 7.9 weeks) and the ORR (28 vs 11.8%) were all improved with lenalidomide compared with investigator's choice [27]. The other two articles were single-arm studies examining the vorinostat + gemcitabine + busulfan/melphalan regimen [18] and fostamatin [12]. Nieto *et al*. concluded that the vorinostat regimen induced high complete response rates (73%) and delivered promising early outcomes in R/R DLBCL patients; 73% of the study population was alive at a median follow-up of 25 months [18]. The study examining fostamatinib found that, although it was generally well tolerated at the 200-mg dose, efficacy was limited, with only 3% of patients achieving any type of response [12].

**Table 3.** **Summary of randomized controlled trial studies examining clinical outcomes of treatment for relapsed or refractory diffuse large B-cell lymphoma.**

**Country/region**	**Design**	**Treatment setting**	**Sample size**	**Follow-up (months)**	**Survival/progression**	**Response**	**Study (year), ref.**	**Source**
USA	Single arm, dose-finding trial	Experimental chemo: vorinostat + gem + busulfan/melphalan	52	Median 25 mos	OS: 73% EFS: 61.5%	ORR: 96% (among DLBCL and measurable disease) CR: 73% (among DLBCL and measurable disease)	Nieto *et al*. (2015), [18]	Full text

UK, USA	Phase II trial	Experimental chemo: fostamatinib	68	NR	NR	ORR: 3% Clinical benefit (ORR + SD): 13%	Flinn *et al*. (2014), [12]	Abstract

NR	Phase II or Phase III	Experimental chemo: lenalidomide vs investigator's choice (gemcitabine, rituximab, etoposide or oxaliplatin)	102	NR	Median OS (weeks) *Overall* LEN: 31 IC: 24.6 *Immunochemistry* LEN GCB: 30 IC GCB: 24.9 LEN non-GCB: 32.3 IC non-GCB: 20.4 *Gene expression profiling* LEN GCB: 30 IC GCB: 20.1 LEN non-GCB: 108.4 IC non-GCB: 18.6 Median PFS (weeks) *Overall* LEN: 13.6 IC: 7.9 *Immunochemistry* LEN GCB: 10.1 IC GCB: 9.0 LEN non-GCB: 15.1 IC non-GCB: 7.1 *Gene expression profiling* LEN GCB: 13.2% IC GCB: 7.1% LEN non-GCB: 82.0% IC non-GCB: 6.2%	ORR *Overall* LEN: 27.5% IC: 11.8% *Immunochemistry* LEN GCB: 26.1% IC GCB: 12.0% LEN non-GCB: 28.6% IC non-GCB: 11.5% *Gene expression profiling* LEN GCB: 21.4% IC GCB: 12.5% LEN non-GCB: 45.5% IC non-GCB: 18.8%	Czuczman *et al*. (2014), [27]	Abstract

France	Phase II trial	Rituximab-based regimens: rituximab + NIMP	50	Median 51.1 mos (among survivors)	Median OS: 28.4 mos Median PFS: 10.5 mos	ORR: 66% (after three cycles) CR: 30% (after three cycles)	Gyan *et al*. (2013), [13]	Full text

Japan, Korea	Phase II study	Rituximab-based regimens: rituximab + bendamustine	59	Median 4.7 mos	Median PFS: 6.7 mos	ORR: 62.7% CR: 37.3%	Ohmachi *et al*. (2013), [19]	Full text

France, Germany, Italy, Spain, UK	Phase II trial	Beam conditioning: conventional BEAM	57	Median 10.5 mos	NR	CR: 84% At 10.5 months, 65% alive and in CR	Isidori *et al*. (2015), [28]	Abstract

BEAM: Etoposide, Ara-C and melphalan; CR: Complete response; DLBCL: Diffuse large B-cell lymphoma; EFS: Event-free survival; GCB: Germinal center B-cell-like; IC: Immunochemistry; LEN: Lenalidomide; mos: Months; NIMP: Vinorelbine, ifosfamide, mitoxantrone and prednisone; NR: Not reported; ORR: Overall response rate; OS: Overall survival; PFS: Progression-free survival; SD: Standard deviation; yrs: Years.

The results of a Phase II clinical trial of rituximab plus vinorelbine, ifosfamide, mitoxantrone and prednisone (R-NIMP) suggested this regimen as a promising option in relapsed DLBCL [13]. The highest OS (median 28.4 months) and PFS (median 10.5 months) outcomes for rituximab-based regimens were reported in this study. Another Phase II study evaluating the BEAM conditioning regimen had the highest rates of complete response (84%) [28] out of all the clinical trials and observational studies in R/R DLBCL included. In a Phase II study of bendamustine plus rituximab (BR) in patients with R/R DLBCL who were not candidates for SCT, an ORR of 63% and a CR rate of 37% was observed, indicating that BR is a promising salvage regimen in DLBCL patients with limited therapeutic options [19].

### Clinical outcomes of treatment for R/R FL

#### Observational study designs

Similar to R/R DLBCL, the majority of the ten observational studies in R/R FL (8/10) focused on either the auto-SCT or allo-SCT treatment settings (Table 4). The majority of studies reported 5-year OS rates from 51 to 72% [16,17,21,23,30,33,37]. This is unsurprising as SCT is commonly regarded as the only curative option in FL. One study found that strong evidence of response based on negative positron emission tomography-computed tomography findings prior to allo-SCT was a strong prognostic factor in patients with relapsing chemosensitive FL [29], with 3-year OS rates of 90% observed in this subset of patients.

**Table 4.** **Summary of observational studies examining clinical outcomes of treatment for follicular lymphoma.**

**Country/region**	**Design**	**Treatment setting**	**Sample size**	**Follow-up (months)**	**Survival/progression**	**Response**	**Study (year), Ref.**	**Source**
France, Germany, Italy, Spain, UK	Retrospective cohort	Auto-SCT vs allo-SCT	875	Median 59 mos (among survivors)	OS *Auto-SCT* 1 yr: 90%; 3 yrs: 78%; 5 yrs: 72% *RIST* 1 yr: 80%; 3 yrs: 68%; 5 yrs: 67% PFS *Auto-SCT* 1 yr: 77%; 3 yrs: 57%; 5 yrs: 48% *RIST* 1 yr: 68%; 3 yrs: 62%; 5 yrs: 57%	NR	Robinson *et al*. 2013, [23]	Full text

France, Germany, Italy, Spain, UK, USA, others	Retrospective cohort	Auto-SCT vs RIC-allo-SCT	518	Median 61 mos	OS *Allo-HCT* 1 yr: 77%; 3 yrs: 69%; 5 yrs: 66% *Auto-HCT* 1 yr: 91%; 3 yrs: 82%; 5 yrs: 74% PFS *Allo-HCT* 1 yr: 70%; 3 yrs: 61%; 5 yrs: 58% *Auto-HCT* 1 yr: 74%; 3 yrs: 51%; 5 yrs: 41%	NR	Klyuchnikov *et al*. (2015), [16]	Full text

France, Germany, Italy, Spain, UK, USA, others	Retrospective cohort	Allo-SCT vs allo-SCT	197	Median Allo-HCT: 57 mos Auto-HCT: 59 mos	OS at 5 yrs Allo-HCT: 59% Auto-HCT: 54% PFS at 5 yrs Allo-HCT: 51% Auto-HCT: 36%	NR	Klyuchnikov *et al*. (2016), [17]	Full text

UK	Retrospective cohort	BEAM-allo vs BEAM-auto-SCT	171	Median BEAM-allo: 7.7 yrs BEAM-auto: 5.5 yrs	OS *BEAM-Allo* 1 yr: 72.5%; 3 yrs: 68.5%; 5 yrs: 66.6%; 10 yrs: 64.4% *BEAM-auto* 1 yr: 90.1%; 2 yrs: 78.7%; 5 yrs: 62.9%; 10 yrs: 48.3% DFS *BEAM-allo* 1 yr: 63%; 3 yrs: 57.4%; 5 yrs: 53.4%; 10 yrs: 48.1% *BEAM-auto* 1 yr: 80.1%; 2 yrs: 64.1%; 5 yrs: 49.9%; 10 yrs: 34.7%	NR	Noriega *et al*. (2014), [33]	Full text

France, Germany, Italy, Spain, UK	Retrospective cohort	RIC-allo-SCT following relapse after auto-SCT	183	Median 59 mos	OS 2 yrs: 63.3%; 5 yrs: 51.1% PFS 2 yrs: 61.8%; 5 yrs: 47.7%	NR	Robinson *et al*. (2016), [22]	Full text

Germany	Retrospective cohort	Allo-SCT after prior chemo	146	Median 9.1 yrs (among survivors)	OS after HCT 1 yr: 67%; 2 yrs: 60%; 5 yrs: 53%; 10 yrs: 48% EFS after HCT 1 yr: 63%; 2 yrs: 53%; 5 yrs: 47%; 10 yrs: 40%	CR: 79%	Heinzelmann *et al*. (2016), [30]	Full text

France	Retrospective cohort	Allo-SCT after response to prior salvage R-chemo	59	Median 37.5 mos	OS at 3 yrs: 90.5% PFS at 3 yrs: 63.1	CR Overall: 72.9% Fludarabine: 75% Aracytine: 78.6% Ifosfamide: 60% PR Overall: 16.9% Fludarabine: 6.3% Aracytine: 14.3% Ifosfamide: 33.3%	Alcantara *et al*. (2015), [29]	Full text

USA	Retrospective cohort	Long-term disease control after allo-SCT (CIBMTR vs EBMT)	1567	Median CIBMTR: 58 mos EBMT: 54 mos	OS at 1 yr: CIBMTR: 74%; EBMT: 75% OS at 3 yr: CIBMTR: 65%; EBMT: 67% OS at 5 yr: CIBMTR: 61%; EBMT: 62% PFS at 1 yr: CIBMTR: 66%; EBMT: 69% PFS at 3 yr: CIBMTR: 56%; EBMT: 58% PFS at 5 yr: CIBMTR: 52%; EBMT: 52%	NR	Sureda *et al*. (2015), [37]	Abstract

NR	Prospective study	W&W vs active therapy	120	Median 64 mos	OS at 5 yrs: 87% No difference between pts initially treated (88%) and W&W patients (87%; p < 0.05) Freedom from treatment failure at 5 yrs: 69% for initially treated and 79% for W&W (p < 0.05)	CR: 60%, among pts with progression and ultimately treated	Solal-Celigny *et al*. (2012), [36]	Full text

Spain	Retrospective cohort	Long-term outcomes after salvage treatment for relapse	283	NR	OS at 10 yrs ICT-sensitive: 83% ICT-refractory: 33% Death ICT-sensitive: 83% ICT-refractory: 50%	NR	Sorigué *et al*. (2015), [38]	Abstract

Auto/allo-HCT: Autologous/allogeneic hematopoietic stem cell transplantation; Auto/allo-SCT: Autologous/allogeneic stem cell transplantation; BEAM: Etoposide, Ara-C and melphalan; CIBMTR: Center for International Blood and Marrow Transplant Research; CR: Complete response; DFS: Disease-free survival; EBMT: European Society for Blood and Marrow Transplantation; EFS: Event-free survival; ICT: Immunochemotherapy; mos: Months; NR: Not reported; OS: Overall survival; PFS: Progression-free survival; PR: Partial response; pts: Patients; RIC: Reduced-intensity conditioning; RIST: Reduced-intensity allo-SCT; SCT: Stem cell transplant; W&W: Watch and wait; yrs: Years.

Several studies compared the outcomes of patients treated with allo-SCT versus auto-SCT with conflicting results, depending upon the subgroup of FL patients included and/or the time period of evaluation [16,17,21]. Klyuchnikov *et al*. found that Grade 1/2 FL patients treated with auto-SCT had superior OS outcomes compared with RIC-allo-SCT (OS: 5-year 74 vs 66%, p = 0.05) [16]. However, in higher-grade patients, no significant difference in OS was observed [17]. In another study by Robinson *et al*., median OS in the auto-SCT arm (59 months) was similar to RIC-allo-SCT (60 months) [23]. With respect to relapse/progression, significantly higher rates at 5 years were observed for allo-SCT vs auto-SCT across the three studies (Klyuchnikov *et al*. (2015) [PFS: 58 vs 41%]; Klyuchnikov *et al*. (2016) [PFS: 51 vs 36%] and Robinson *et al*. [PFS: 57 vs 48%]) [16,17,23]. Finally, in another study comparing BEAM-auto-SCT with BEAM-allo-SCT in 171 patients with relapsed FL [33], OS and PFS were not statistically different at any of the time points examined. For the subset of patients in complete response at transplant, however, BEAM-allo seemed to show improved DFS at 10 years (71.4 vs 27.7%, p = 0.056).

One study compared outcomes among patients receiving a watch and wait strategy versus initial therapy with rituximab in low tumor-burden FL, a favorable prognostic subgroup. No difference in OS at 5 years was observed between groups (watch and wait strategy 87% vs initial therapy 88%). Upon disease progression and receiving active treatment, 60% of evaluable patients achieved complete response. Finally, a study by Sorigué *et al*. provided further evidence of the positive impact of therapy response on long-term outcomes in FL patients treated with prior first-line rituximab-based immunochemotherapy [38]. Of those patients who responded initially to primary therapy and later, upon relapse, were treated with salvage therapy, 83% were still alive at 10 years compared with only 33% of treatment-refractory patients.

#### Clinical trials

There were five studies reporting on R/R FL treatment trials (Table 5). Three studies examining the efficacy of experimental chemotherapy regimens were identified, with two providing evidence supporting obinutuzumab in R/R FL [34,35]. In these two trials of obinutuzumab regimens, complete response ranged from 6% for minimal residual disease (MRD)-positive patients treated with obinutuzumab plus bendamustine [34] to 50% among patients positive for the CD20 antigen (CD20^+^) receiving obinutuzumab + fludarabine and cyclophosphamide (FC) [35]. This Phase Ib study by Radford *et al*. also noted very high ORRs of 93–96% following induction with study treatments (obinutuzumab + FC or obinutuzumab + CHOP). These results are in contrast with Ganjoo *et al*., who assessed ocaratuzumab in a similarly enrolled CD20^+^ FL population [39]]. They found ORRs around 30%, and recommended further studies to establish a role for ocaratuzumab in CD20^+^ FL. Finally, Pott *et al*. specifically investigated the prognostic relevance of MRD in patients treated with obinutuzumab, and found evidence suggesting that the treatment was more effective among patients with MRD-negative status versus positive (complete response rate of 28 and 6%, respectively) [34]. The study also indicated greater depth of response with obinutuzumab plus bendamustine over bendamustine alone, with nearly twice the patients achieving MRD-negative status with obinutuzumab plus bendamustine (82 vs 43%; p < 0.0001) during induction.

**Table 5.** **Summary of randomized controlled trial studies examining clinical outcomes of treatment for follicular lymphoma.**

**Country/region**	**Design**	**Treatment setting**	**Sample size**	**Follow-up (months)**	**Survival/progression**	**Response**	**Study (year), ref.**	**Source**
USA	Phase II, single-arm study	Allo-SCT after prior chemo	62	Median 47 mos (among survivors)	OS 2 yrs: 84% 3 yrs: 82% PFS 2 yrs: 73% 3 yrs: 71%	ORR: 94% CR: 90%	Laport *et al*. (2016), [32]	Full text

France, Germany, Italy, Spain, UK	Phase Ib trial	Experimental chemo: obinutuzumab + CHOP vs obinutuzumab + FC	56	NR	NR	ORR at end of induction CHOP: 96% FC: 93% CR at end of induction CHOP: 39% FC: 50%	Radford *et al*. (2013), [35]	Full text

USA	Phase I or Phase II study	Experimental chemo: ocaratuzumab	50	NR	Median PFS Investigator Assessment: 38 wks Central Assessment: 91 wks	ORR: 30% CR: 8 patients	Ganjoo *et al*. (2015), [39]	Full text

Canada, France, Germany, Italy, Spain, UK, USA	Open-label, Phase III study	Experimental chemo: obinutuzumab + bendamustine (G + B)	321	NR	NR	CR MRD positive: 6% MRD negative: 28% MRD negative: G plus B 82 vs B 43%, p < 0.0001)	Pott *et al*. (2015), [34]	Abstract

	Phase II trial	90Y-IT following R-based chemo	50	Median 5 yrs	Median OS: 75.2 mos OS at 5 yrs: 77.5% Median PFS: 23.1 mos PFS at 1 yr: 94.1% PFS at 3 yrs: 34.0% PFS at 5 yrs: 21.9%	ORR Week 8: 94.2% Week 18: 98% CR Week 8: 7.7% Week 18: 30%	Illidge *et al*. (2016), [31]	Full text

90Y-IT: 90Y-ibritumomab-tiuxetan; Auto/allo-SCT: Autologous/allogeneic stem cell transplantation; CHOP: Cyclophosphamide, doxorubicin, vincristine and prednisone; CR: Complete response; FC: Fludarabine and cyclophosphamide; mos: Months; MRD: Minimal residual disease; NR: Not reported; ORR: Overall response rate; OS: Overall survival; PFS: Progression-free survival; wks: Weeks; yrs: Years.

Illidge *et al*. evaluated the effectiveness of 90Y-IT in CD20^+^-relapsed patients receiving prior immunochemotherapy with rituximab [31]. After 2 months of treatment with 90Y-IT, an ORR of 98% was achieved, with 30% of the patients achieving complete response/CRu. Median OS was 75.2 months; 1- and 3-year PFS rates were 94.1 and 34.0% respectively. Only one study examined allo-SCT in patients experiencing relapse or progression after prior chemotherapy [32]. Results indicate that allo-SCT offers durable, long-term remissions for relapsed patients. The 2- and 3-year OS rates in patients who were chemosensitive undergoing RIC-allo-SCT were 84 and 82%, respectively. Similarly, the 2- and 3-year PFS rates were 73 and 71%, respectively. These findings further support allo-SCT prolongation of survival and time to relapse in chemosensitive patients.

### Economic outcomes of treatment

Among the studies included in this review, only one was identified that reported data on hospitalizations. No study presented evidence on medical costs or hospitalizations in R/R FL. Gyan *et al*. reported a hospitalization rate of 20% due to toxicity following treatment with R-NIMP among CD20^+^ DLBCL patients at first relapse. Patients were hospitalized for a median length of stay of 6 days [13]. The most common safety events were Grade 3–4 toxicities for neutrophils (50%) and platelets (15%).

## Discussion

The objective of this work was to comprehensively review available data on the clinical effectiveness associated with different therapies for the treatment of R/R DLBCL and R/R FL. Although more than 2000 unique citations were identified, only 33 articles examined therapeutic effectiveness in patients with R/R DLBCL or R/R FL. Although some RCTs were included in this review, the primary objective was to identify real-world evidence; thus, not all trials were systematically identified. Furthermore, most of the RCTs included in this review were Phase I or Phase II studies, which generally have fewer enrolled patients when compared with Phase III studies for these diseases. Several of the larger RCTs that examined rituximab-based regimens in this population, including CORAL [40], the Canadian Cancer Trials Group LY.12 [41] and ORCHARRD [42], were not captured within the search algorithm due to either publication date or indexing. As a result of the design of this review, observational studies made up the majority of data included.

Sample sizes were generally higher for observational studies, which are consistent with this type of study design. A sample size of at least 50 patients was required for inclusion in the review to ensure the robustness of the data identified. While observational studies may have more potential to introduce selection bias, the majority of observational studies in this analysis sought to address this by incorporating multivariate analysis methods. Additionally, observational studies provide their own value as they demonstrate the performance of therapies used in standard clinical practice, making the results more easily generalized to the typical patient. These findings often complement and/or supplement data from clinical trials.

Overall, our review showed that in the past 5 years, evidence generation for R/R DLBCL treatment effectiveness has been limited, with studies focusing predominantly on auto- versus allo-SCT, followed by chemotherapeutic approaches, consisting of both experimental and rituximab-based combination therapies. This is not surprising, as SCT has long been considered the optimal salvage strategy following initial relapse in DLBCL (European Society for Medical Oncology Guidelines) [43,44]. While one of the highest OS rates observed (71% at 39 months) was reported in patients treated with auto-SCT [9], there have been numerous advancements in supportive care for allo-SCT over the last decade, including improvements in donor-selection tools and RIC regimens [45–47]. These developments, along with a lack of clarity around potential deterioration of outcomes for auto-SCT in the era of immunochemotherapy, prompted Robinson *et al*. to conduct a study comparing different SCT treatment strategies [21]. Despite these changes, the authors observed continued greater OS and nonrelapse mortality with auto-SCT compared with allo-SCT. Within the SCT setting, longer-term survival rates were variable per study and ranged from a 3-year OS of 18% to a 4-year OS of 64%. This variability is likely a result of heterogeneity within DLBCL, as the 3-year 18% OS was based on a subgroup of patients with germinal center B-cell-like DLBCL, whereas the 4-year 64% OS calculation was based on a population that was negative for circulating tumor disease. Response rates for SCT were reported in only one R/R DLBCL study examining allo-SCT after prior failure with auto-SCT, with an ORR of 49% [20]. Overall, the findings of this review also indicate a high degree of variability across different DLBCL subpopulations, supporting the need for further study and use of predictive biomarkers to identify at-risk populations who may benefit from novel treatment strategies.

For DLBCL patients experiencing relapse or progression, treatment guidelines recommend high-dose chemotherapy coupled with SCT, with rituximab combination regimens commonly used for patients ineligible for SCT [44]. Over 70% of the studies included in this review examined these two treatment regimens – unsurprising as they are the dominant first-line treatments for these groups. With regard to findings pertaining to immunochemotherapy in R/R DLBCL, the prognosis among patients receiving rituximab-based regimens was inferior to SCT, with median OS statistics ranging from 10.8 to 28.4 months. Not surprisingly, the lowest OS and PFS outcomes were observed in studies that exclusively focused on patients ineligible for auto-SCT due to age or co-morbidity [7,19]. The median age range (64–76 years) also tended to be older for studies examining rituximab-based regimens, with a higher proportion of patients having more advanced disease (61–76% stage 3 or 4) relative to studies in the SCT setting. Five-year OS rates for BEAM conditioning regimens (69–86%) were among the highest of all the studies included in this review, with one study evaluating different conditioning regimens showing a slight 5-year OS advantage (but no difference in DFS) for Z-BEAM over BEAM/R. BEAM response rates were not frequently reported, but the highest response rate (84%) was observed in a Phase II study evaluating BEAM conditioning regimen.

Among the RCTs included in this study, most were focused on survival outcomes for experimental chemotherapeutic regimens. The highest OS reported among these studies was a median of 28.4 months for a rituximab-based regimen. This particular study also reported the highest median PFS of 10.5 months. While these median survival times are higher than what was reported for observational studies, this may have been due to age or performance status differences between the study populations. Six of the included clinical trials reported response outcomes, with the highest being an ORR of 96% in a study of vorinostat, gemcitabine and busulfan/melphalan [18].

A recently published patient-level analysis [48] pooled the results of two pivotal rituximab trials (CORAL and CCTG LY.12) with two large observational cohorts to examine response rates and survival for patients with refractory DLBCL. Pooled results showed a 26% ORR with a range of 20–31% among the four included studies. Findings for pooled OS were similar: median OS of 6.3 months (range of 5.9–7.0 months among included studies; 20% of patients alive at 2 years). The results of this retrospective analysis are significant for several reasons: the authors concluded that, overall, there are limited data published for this patient population, which is confirmatory to the findings of the current review. Even with the current treatment landscape including multiple rituximab-based therapies, response and survival outcomes for refractory DLBCL remain poor. The rate of response and median survival was similar across study designs.

Similar to R/R DLBCL, there were limited data on treatment-related effectiveness outcomes reported for R/R FL in the past 5 years. Consistent with its more indolent pathophysiology, survival outcomes for R/R FL were generally better than those reported for R/R DLBCL.

Approximately half of studies focusing on R/R FL examined outcomes for auto-SCT or allo-SCT. This was surprising as treatment guidelines suggest reserving SCT for only select cases [44]. Five-year OS with SCT ranged from 51 to 72%. There was some evidence indicating that survival is improved with auto-SCT versus RIC-allo-SCT, although this may have been attributable to differences in disease stage at diagnosis and was not consistently observed in the available literature. As with R/R DLBCL, data on treatment response rates in the SCT setting were limited in observational studies, more prevalent among RCTs, but were overall quite variable across studies and patient subgroups.

According to treatment guidelines, chemotherapy regimens containing rituximab should be the mainstay of treatment for R/R FL [43], as NCCN guidelines currently recommend SCT only for patients who progress within the first 6 months of first-line maintenance with rituximab, or for patients on their second or third remission. When examining studies focused on chemotherapy regimens, the highest ORR (96%) was reported in a Phase IB trial for patients receiving obinutuzumab in combination with CHOP [35]. ORR ranged from 30–90%, and complete response ranged from 6% for MRD-positive patients receiving obinutuzumab to 50% for CD20^+^ patients receiving obinutuzumab with FC. In contrast, the data for the experimental agent ocaratuzumab were not as robust, with an ORR of only around 30% in patients with R/R CD20^+^ FL. As with DLBCL, patient populations of studies focusing on the SCT setting were generally younger compared with those treated with chemotherapy.

As with all systematic literature reviews, there are certainly limitations in the scope of what the review can address. The objective of this SLR was to identify studies of patients with R/R DLBCL or R/R FL, although the search allowed for inclusion of studies including all DLBCL or FL patients. Patient demographics, such as age, were different among patients who received stem cell therapies and those who received other treatments, making it inherently difficult to truly assess the effectiveness of each treatment on clinical outcomes. As discussed earlier, in studies included for R/R FL, SCT was the most prevalent therapy assessed, which is in contrast to what guidelines would suggest. Given the higher reporting of survival outcomes observed in the studies evaluating SCT, it is possible that some bias toward these studies may have been introduced by the search strategy's focus on effectiveness outcomes. Finally, this review did not address newer therapies, including chimeric antigen receptor T-cell therapies, gene treatments for R/R DLBCL. Axicabtagene ciloleucel gained the US FDA approval in October of 2017 for large B-cell lymphoma (including DLBCL) after failure with at least two other therapy types; however, studies examining this treatment were not published until after the review's timeframe. Similarly, tisagenlecleucel gained approval from the FDA in early May 2018 for the treatment of R/R DLBCL patients. Both of these treatments could have significant impact on the treatment landscape. Axicabtagene ciloleucel showed a complete response rate of 54% in a multinational clinical trial [49] and tisagenlecleucel treatment showed an ORR of 50% (32% complete response) in a pivotal Phase II trial [50].

## Conclusion

Overall, the results presented here indicate that there are limited data regarding real-world effectiveness and economic outcomes for approved treatment strategies and clinical outcomes in patients with R/R DLBCL or R/R FL. Among studies analyzed for R/R DLBCL, treatment courses ranged from SCT to combination chemotherapy, although most of the studies examined SCT. Survival rates and treatment responses were variable and survival outcomes were more likely to be reported in studies that examined SCT as a treatment modality, whereas studies examining experimental regimens were more likely to report response outcomes. Among studies analyzed for R/R FL, treatment settings ranged from SCT to chemotherapy, including experimental types and 90Y-IT. Survival outcomes were more often reported in studies evaluating SCT, while information on response was limited in these SCT studies, and survival rates were variable. Response rates in patients who received experimental chemotherapy were also variable. This variability in survival and response rates is likely a consequence of heterogeneity within DLBCL and FL populations, and suggests a need for further research on prognostic biomarkers to guide therapy choice in R/R DLBCL and FL patients. For both DLBCL and FL, SCT-based treatment strategies were the most analyzed in this review. However, SCT can be used in only a subset of patients, as it is not currently recommended for the elderly or those with multiple comorbidities. These findings suggest that treatment options for patients ineligible for SCT remain a significant unmet need.

## Future perspective

Many of the current studies of DLBCL and FL included in this review examined SCT. Indeed, the studies suggest that patients treated with this strategy have the best prognosis in terms of both OS and overall response. However, SCT can be used in only a subset of patients, as it is not currently recommended for the elderly or those with multiple co-morbidities. A lack of viable treatment options with proven long-term results for these patients currently represents a significant gap in care. Further research into nontransplant options is likely to gain momentum in order to fill this critical unmet need, along with a push to identify predictive biomarkers to distinguish the patients who are most likely to benefit from these novel treatment strategies. Additionally, given the positive long-term results associated with SCT, we are likely to see more prognostic studies further delineating the specific subsets of patients who are the best candidates for this therapy. Lastly, RIC regimens for allo-SCT have already reduced the high treatment-related mortality historically associated with this procedure, so we expect additional research into these regimens in an effort to expand the numbers of patients with R/R DLBCL and R/R FL who can benefit from SCT.

**Executive summary**

- Non-Hodgkin lymphoma (NHL) is the most prevalent hematological malignancy. Diffuse large B-cell lymphoma (DLBCL) and follicular lymphoma (FL) are the most common types of NHL, accounting for approximately 65% of all NHLs.
- A frequently used regimen for both indications is rituximab given in combination with the regimen of cyclophosphamide, doxorubicin, vincristine and prednisone (R-CHOP).
- R-CHOP significantly improves survival in FL and DLBCL.
- Approximately a third of DLBCL patients develop relapsed or refractory (R/R) disease. Although FL is a more indolent form of NHL, there is currently no cure and relapses occur.
- Current therapeutic options for patients include stem cell transplant (SCT) and chemotherapy/immunotherapy. More effective therapies for patients ineligible for SCT remain an important objective.
- This review systematically evaluated the evidentiary base for therapies used in R/R DLBCL or R/R FL.
- There was limited evidence on effectiveness outcomes for R/R DLBCL and R/R FL in the past 5 years. Treatments included SCT and chemotherapy. No study of R/R FL examined rituximab-based chemotherapy, despite treatment guidelines describing it as a treatment mainstay.
- The majority of observational studies included for R/R DLBCL examined the effectiveness of either autologous (auto-) SCT or allogeneic (allo-) SCT. One of the highest overall survival (OS) rates observed (71% at 39 months) was reported in patients treated with auto-SCT; this mode of therapy is considered the optimal salvage strategy following initial relapse.
- For DLBCL, the prognosis among those receiving rituximab-based regimens was inferior to SCT, with median OS from 10.8 to 28.4 months. The lowest OS and progression-free survival were observed in studies that exclusively focused on patients ineligible for auto-SCT due to age or co-morbidity.
- High-dose chemotherapy followed by auto-SCT is a well-defined treatment modality for R/R DLBCL. Five-year OS rates for etoposide, Ara-C and melphalan (BEAM) conditioning regimens ranged from 69 to 86% and were among the highest reported in the review.
- Approximately half of FL studies examined outcomes for auto-SCT or allo-SCT, which was surprising as treatment guidelines suggest reserving SCT for only select cases. 5-year OS ranged from 51 to 72%. Some evidence indicates that survival is improved with auto-SCT versus reduced-intensity conditioning-allo-SCT, although this may have been attributable to differences in baseline disease stage and was not consistently observed in the available literature.
- Experimental chemotherapy regimens were examined in several studies of FL. The highest overall response rate (96%) was reported in a Phase IB trial examining obinutuzumab in combination with CHOP. In contrast, data for the experimental agent ocaratuzumab reported an overall response rate of only 30% in CD20^+^ patients.

## References

[1] Armitage JO, Gascoyne RD, Lunning MA, Cavalli F (2017). Non-Hodgkin lymphoma. *Lancet*.

[2] Nowakowski GS, Chiappella A, Witzig TE (2016). ROBUST: lenalidomide-R-CHOP versus placebo-R-CHOP in previously untreated ABC-type diffuse large B-cell lymphoma. *Future Oncol.*.

[3] National Comprehensive Cancer Network (2017). NCCN Clinical Practice Guidelines in Oncology (NCCN Guidelines^®^) B-cell Lymphomas, version 7.2017. http://www.nccn.org/professionals/physician_gls/pdf/b-cell.pdf.

[4] Zhou Z, Sehn LH, Rademaker AW (2014). An enhanced International Prognostic Index (NCCN-IPI) for patients with diffuse large B-cell lymphoma treated in the rituximab era. *Blood*.

[5] PDQ^®^ Adult Treatment Editorial Board (2017). Adult non-Hodgkin lymphoma treatment (PDQ^®^)–health professional version. http://www.cancer.gov/types/lymphoma/hp/adult-nhl-treatment-pdq.

[6] Amin AD, Peters TL, Li L (2017). Diffuse large B-cell lymphoma: can genomics improve treatment options for a curable cancer?. *Cold Spring Harb. Mol. Case Stud.*.

[7] Arcari A, Chiappella A, Spina M (2015). Safety and efficacy of rituximab plus bendamustine in relapsed or refractory diffuse large B-cell lymphoma patients: an Italian retrospective multicenter study. *Leuk. Lymphoma.*.

[8] Avivi I, Canals C, Vernant JP (2014). Matched unrelated donor allogeneic transplantation provides comparable long-term outcome to HLA-identical sibling transplantation in relapsed diffuse large B-cell lymphoma. *Bone Marrow Transplant.*.

[9] Avivi I, Boumendil A, Finel HH (6–9 December 2014). Autologous stem cell transplantation for primary mediastinal B cell lymphoma in the rituximab era: a retrospective study by the EBMT Lymphoma Working Party. *American Society of Hematology (ASH) 56th Annual Meeting and Exposition*.

[10] Farooq F, Maurer MJ, Ansell SM (5–8 December 2015). Treatment patterns and outcomes of DLBCL after failure of front-line immunochemotherapy. *American Society of Hematology (ASH) 57th Annual Meeting and Exposition*.

[11] Fenske TS, Ahn KW, Graff TM (2016). Allogeneic transplantation provides durable remission in a subset of DLBCL patients relapsing after autologous transplantation. *Br. J. Haematol.*.

[12] Flinn IW, van der Jagt R, Kahl BS (2014). Randomized trial of bendamustine-rituximab or R-CHOP/R-CVP in first-line treatment of indolent NHL or MCL: the BRIGHT study. *Blood*.

[13] Gyan E, Damotte D, Courby S (2013). High response rate and acceptable toxicity of a combination of rituximab, vinorelbine, ifosfamide, mitoxantrone and prednisone for the treatment of diffuse large B-cell lymphoma in first relapse: results of the R-NIMP GOELAMS study. *Br. J. Haematol.*.

[14] Herrera AF, Mei MG, Low L (5–8 December 2015). Double expressing (MYC/BCL2) and double-hit diffuse large B-cell lymphomas have inferior survival following autologous stem cell transplantation. *American Society of Hematology (ASH) 57th Annual Meeting and Exposition*.

[15] Khouri I, Sui D, Turturro F (5–8 December 2015). *In-vivo* purging with rituximab (R) followed by Z/BEAM vs BEAM/R autologous stem cell conditioning for relapsed diffuse large B-cell lymphoma (DLBCL) patients (pts): mature results from a combined analysis of 3 trials. *American Society of Hematology (ASH) 57th Annual Meeting and Exposition*.

[16] Klyuchnikov E, Bacher U, Kröger NM (2015). Reduced-intensity allografting as first transplantation approach in relapsed/refractory Grades one and two follicular lymphoma provides improved outcomes in long-term survivors. *Biol. Blood Marrow Transplant.*.

[17] Klyuchnikov E, Bacher U, Woo Ahn K (2016). Long-term survival outcomes of reduced-intensity allogeneic or autologous transplantation in relapsed Grade 3 follicular lymphoma. *Bone Marrow Transplant.*.

[18] Nieto Y, Valdez BC, Thall PF (2015). Vorinostat combined with high-dose gemcitabine, busulfan, and melphalan with autologous stem cell transplantation in patients with refractory lymphomas. *Biol. Blood Marrow Transplant.*.

[19] Ohmachi K, Niitsu N, Uchida T (2013). Multicenter Phase II study of bendamustine plus rituximab in patients with relapsed or refractory diffuse large B-cell lymphoma. *J. Clin. Oncol.*.

[20] Rigacci L, Puccini B, Dodero A (2012). Allogeneic hematopoietic stem cell transplantation in patients with diffuse large B cell lymphoma relapsed after autologous stem cell transplantation: a GITMO study. *Ann. Hematol.*.

[21] Robinson SP, Boumendil A, Finel H (2016). Autologous stem cell transplantation for relapsed/refractory diffuse large B-cell lymphoma: efficacy in the rituximab era and comparison to first allogeneic transplants. A report from the EBMT Lymphoma Working Party. *Bone Marrow Transplant.*.

[22] Robinson SP, Boumendil A, Finel H (2016). Reduced intensity allogeneic stem cell transplantation for follicular lymphoma relapsing after an autologous transplant achieves durable long term disease control. An analysis from the Lymphoma Working Party of the EBMT. *Ann. Oncol.*.

[23] Robinson SP, Canals C, Luang JJ (2013). The outcome of reduced intensity allogeneic stem cell transplantation and autologous stem cell transplantation when performed as a first transplant strategy in relapsed follicular lymphoma: an analysis from the Lymphoma Working Party of the EBMT. *Bone Marrow Transplant.*.

[24] Rovira J, Valera A, Colomo L (2015). Prognosis of patients with diffuse large B cell lymphoma not reaching complete response or relapsing after frontline chemotherapy or immunochemotherapy. *Ann. Hematol.*.

[25] Lisenko K, McClanahan F, Schoning T (2016). Minimal renal toxicity after Rituximab DHAP with a modified cisplatin application scheme in patients with relapsed or refractory diffuse large B-cell lymphoma. *BMC Cancer*.

[26] Khouri I, Saliba R, Xu-Monette Z (2014). Outcomes following allogeneic stem cell transplantation (alloSCT) in patients with primary mediastinal (PMBL), germinal center B (GCB) and non-GCB cell-like diffuse large B cell lymphomas (DLBCL). Presented at: *American Society of Hematology (ASH) 56th Annual Meeting and Exposition*, 6–9 December 2014, San Francisco, CA. *Blood*.

[27] Czucman MS, Davis A, Linton KM (6–9 December 2014). Phase 2/3 multicenter, randomized study comparing the efficacy and safety of lenalidomide versus investigator's choice in relapsed/refractory DLBCL. *American Society of Hematology (ASH) 56th Annual Meeting and Exposition*.

[28] Isidori A, Guidi S, Scalzulli PR (5–8 December 2015). Benda-beam high-dose therapy prior to auto-SCT is effective in resistant/relapsed DLBCL. *American Society of Hematology (ASH) 57th Annual Meeting and Exposition*.

[29] Alcantara M, Dupuis J, Mareschal S (2015). PET/CT before autologous stem cell transplantation predicts outcome in refractory/relapsed follicular lymphoma. *Eur. J. Nucl. Med. Mol. Imaging*.

[30] Heinzelmann F, Bethge W, Beelen DW (2016). Allogeneic hematopoietic cell transplantation as curative therapy for non-transformed follicular lymphomas. *Bone Marrow Transplant.*.

[31] Illidge TM, McKenzie HS, Mayes S (2016). Short duration immunochemotherapy followed by radioimmunotherapy consolidation is effective and well tolerated in relapsed follicular lymphoma: 5-year results from a UK National Cancer Research Institute Lymphoma Group study. *Br. J. Haematol.*.

[32] Laport GG, Wu J, Logan B (2016). Reduced intensity conditioning with fludarabine, cyclophosphamide, and high dose rituxan for allogeneic hematopoietic cell transplantation for follicular lymphoma: a Phase II multicenter trial from the Blood and Marrow Transplant Network (BMT CTN). *Biol. Blood Marrow Transplant.*.

[33] Noriega V, Kaur H, Devereux S (2014). Long term follow-up of BEAM-autologous and BEAM-alemtuzumab allogeneic stem cell transplantation in relapsed advanced stage follicular lymphoma. *Leuk. Res.*.

[34] Pott C, Belada D, Danesi N (5–8 December 2015). Analysis of minimal residual disease in follicular lymphoma patients in gadolin, a Phase III study of obinutuzumab plus bendamustine versus bendamustine in relapsed/refractory indolent non-Hodgkin lymphoma. *American Society of Hematology (ASH) 57th Annual Meeting and Exposition*.

[35] Radford J, Davies A, Cartron G (2013). Obinutuzumab (GA101) plus CHOP or FC in relapsed/refractory follicular lymphoma: results of the GAUDI study (BO21000). *Blood*.

[36] Solal-Ceĺigny P, Bellei M, Marcheselli L (2012). Watchful waiting in low-tumor burden follicular lymphoma in the rituximab era: results of an F2-study database. *J. Clin. Oncol.*.

[37] Sureda A, Zhang MJ, Dreger P (5–8 December 2015). Allogeneic stem cell transplantation for relapsed/refractory (R/R) follicular lymphoma (FL). A joint study between the European Society for Blood and Marrow Transplantation (EBMT) and the Center for International Blood and Marrow Transplant Research (CIBMTR). *American Society of Hematology (ASH) 57th Annual Meeting and Exposition*.

[38] Sorigué M, Sancho JM, Mercadal S (5–8 December 2015). Prevalence, predictive factors therapy and outcome of patients with follicular lymphoma refractory to first line immunochemotherapy. *American Society of Hematology (ASH) 57th Annual Meeting and Exposition*.

[39] Ganjoo KN, de Vos S, Pohlman BL (2015). Phase 1/2 study of ocaratuzumab, an Fc-engineered humanized anti-CD20 monoclonal antibody, in low-affinity Fc-gamma-RIIIa patients with previously treated follicular lymphoma. *Leuk. Lymphoma*.

[40] Gisselbrecht C, Glass B, Mounier N (2010). Salvage regimens with autologous transplantation for relapsed large B-cell lymphoma in the rituximab era. *J. Clin. Oncol.*.

[41] Crump M, Kuruvilla J, Couban S (2014). Randomized comparison of gemcitabine, dexamethasone, and cisplatin versus dexamethasone, cytarabine, and cisplatin chemotherapy before autologous stem-cell transplantation for relapsed and refractory aggressive lymphomas: NCIC-CTG LY.12. *J. Clin. Oncol.*.

[42] van Imhoff GW, McMillan A, Matasar MJ (2017). Ofatumumab versus Rituximab salvage chemoimmunotherapy in relapsed or refractory diffuse large B-cell lymphoma: the ORCHARRD study. *J. Clin. Oncol.*.

[43] Dreyling M, Ghielmini M, Rule S (2016). Newly diagnosed and relapsed follicular lymphoma: ESMO Clinical Practice Guidelines for diagnosis, treatment and follow-up. *Ann. Oncol.*.

[44] Tilly H, Gomes da Silva M, Vitolo U (2015). Diffuse large B-cell lymphoma (DLBCL): ESMO Clinical Practice Guidelines for diagnosis, treatment and follow-up. *Ann. Oncol.*.

[45] Rezvani AR, Storer B, Maris M (2008). Nonmyeloablative allogeneic hematopoietic cell transplantation in relapsed, refractory, and transformed indolent non-Hodgkin's lymphoma. *J. Clin. Oncol.*.

[46] Sirvent A, Dhedin N, Michallet M (2010). Low nonrelapse mortality and prolonged long-term survival after reduced-intensity allogeneic stem cell transplantation for relapsed or refractory diffuse large B cell lymphoma: report of the Societe Francaise de Greffe de Moelle et de Therapie Cellulaire. *Biol. Blood Marrow Transplant.*.

[47] Thomson KJ, Morris EC, Bloor A (2009). Favorable long-term survival after reduced-intensity allogeneic transplantation for multiple-relapse aggressive non-Hodgkin's lymphoma. *J. Clin. Oncol.*.

[48] Crump M, Neelapu SS, Farooq U (2017). Outcomes in refractory diffuse large B-cell lymphoma: results from the international SCHOLAR-1 study. *Blood*.

[49] Neelapu SS, Locke FL, Bartlett NL (2017). Axicabtagene ciloleucel CAR T-cell therapy in refractory large B-cell lymphoma. *N. Engl. J. Med.*.

[50] Schuster SJ, Bishop MR, Tam CS (9–12 December 2017). Primary analysis of Juliet: a global, pivotal, Phase 2 trial of CTL019 in adult patients with relapsed or refractory diffuse large B-cell lymphoma. *American Society of Hematology (ASH) 59th Annual Meeting and Exposition*.

